# Differential inhibition of polymorphonuclear leukocyte recruitment *in vivo* by dextran sulphate and fucoidan

**DOI:** 10.1155/S0962935196000506

**Published:** 1996-10

**Authors:** N. Van Osselaer, M. Rampart, A. G. Herman

**Affiliations:** 1 Division of Pharmacology Faculty of Medicine University of Antwerp (UIA) Universiteitsplein 1 Wilrijk B-2610 Belgium

## Abstract

The selectin-mediated rolling of leukocytes along the endothelial cells is a prerequisite step followed by firm adhesion and extravasation into the inflamed tissue. This initial contact can be suppressed by sulphated polysaccharides. We have studied the effect of sulphated polysaccharides on the ultimate polymorphonuclear leukocyte (PMN) recruitment and plasma leakage in rabbit skin in response to intradermal injection of various inflammatory mediators. PMN infiltration evoked by various PMN chemoattractants (FMLP, C5a desArg, LTB_4_ and IL-8) was significantly inhibited after intravenous injection of dextran sulphate (25 mg/kg), heparin (2 × 90 mg/kg) or fucoidan (1 mg/kg). PMN-dependent plasma leakage was equally well reduced by the different sulphated polymers. Vascular permeability induced by histamine or thrombin acting via a PMN-independent mechanism was not reduced. Fucoidan was the only polysaccharide able to suppress IL-1-induced PMN infiltration for 60–70%. Local administration of dextran sulphate had no effect on PMN-dependent plasma leakage. Differential inhibition of PMN recruitment was determined after injection of dextran sulphate or fucoidan depending on the type of insult. Therefore, these results suggest that different adhesion pathways are utilized during PMN recruitment *in vivo* in response to chemoattractants and IL-1.


Mediators of Inflammation, 5, 346–357 (1996)

### Differential inhibition of polymorphonuclear leukocyte recruitment *in vivo* by dextran sulphate and fucoidan

N. Van Osselaer,[CA] M. Rampart† and A. G. Herman

Division of Pharmacology, Faculty of Medicine, University of Antwerp (UIA), Universiteitsplein 1, B-2610 Wilrijk, Belgium
†Deceased

[CA]Corresponding Author
Tel: (+32) 3 820 26 36
Fax: (+32) 3 820 25 67

**THE selectin-mediated rolling of leukocytes along the endothelial cells is a prerequisite step followed by firm adhesion and extravasation into the inflamed tissue. This initial contact can be suppressed by sulphated polysaccharides. We have studied the effect of sulphated polysaccharides on the ultimate polymorphonuclear leukocyte (PMN) recruitment and plasma leakage in rabbit skin in response to intradermal injection of various inflammatory mediators. PMN infiltration evoked by various PMN chemoattractants (FMLP, C5a desArg, $LTB_4$ and IL-8) was significantly inhibited after intravenous injection of dextran sulphate (25 mg/kg), heparin (2 × 90 mg/kg) or fucoidan (1 mg/kg). PMN-dependent plasma leakage was equally well reduced by the different sulphated polymers. Vascular permeability induced by histamine or thrombin acting via a PMN-independent mechanism was not reduced. Fucoidan was the only polysaccharide able to suppress IL-1-induced PMN infiltration for 60–70%. Local administration of dextran sulphate had no effect on PMN-dependent plasma leakage. Differential inhibition of PMN recruitment was determined after injection of dextran sulphate or fucoidan depending on the type of insult. Therefore, these results suggest that different adhesion pathways are utilized during PMN recruitment *in vivo* in response to chemoattractants and IL-1.**

**Key words:** Dextran sulphate, Fucoidan, Heparin, Inflammation, Oedema formation, PMN recruitment, Rabbit

### Introduction

An inflammatory reaction (e.g. adult respiratory distress syndrome, ischaemic reperfusion injury or arthritis) is characterized by leukocyte emigration and plasma protein leakage from the blood stream into the extravascular injured tissue. Leukocyte recruitment is designed as a multistep process in which several types of cell adhesion molecules are involved.[1,2] The initial contact of leukocytes with postcapillary endothelial cells, described as rolling, is mediated by the selectin family of adhesion molecules.[3,4] The subsequent firm adhesion and transmigration through the endothelial cell lining into the inflamed tissue is mediated by leukocyte $\beta_2$-integrins (CD11/CD18) and endothelial immunoglobulin-like molecules (ICAM-1 and PECAM-1).[5–7]

The selectin family consists of three closely related cell surface molecules: L-selectin (CD62L, MEL-14, LAM-1), P-selectin (CD62P, GMP-140) and E-selectin (CD62E, ELAM-1). L-selectin, originally described as a lymphocyte homing receptor (MEL-14)[8] is constitutively present on PMNs, monocytes and most of the lymphocytes. PMN activation results in a rapid removal of L-selectin from the cell surface ('shedding').[9] P-selectin normally exists preformed in $\alpha$-granules of platelets and in Weibel-Palade bodies of endothelial cells. After stimulation with histamine, thrombin or oxygen radicals P-selectin is mobilized within minutes on the endothelial cell surface.[10] E-selectin is expressed on endothelial cells via *de novo* mRNA and protein synthesis after stimulation with various inflammatory agents, i.e. LPS, IL-1 and TNF.[11] *In vitro* and *in vivo* studies have shown that leukocyte L-selectin as well as endothelial P- and E-selectin are involved in leukocyte rolling supporting the final leukocyte recruitment.[9,12–15]

© 1996 Rapid Science Publishers

A common characteristic for the three selectins is their weak binding via their extracellular $Ca^{2+}$-dependent (C-type) lectin domain to sialylated, fucosylated carbohydrates, like the tetrasaccharide sialyl Lewis X ($SLe^x$).[16] Nevertheless high affinity glycoprotein ligands for all three selectins have been described.[4] This suggests that *in vivo* each selectin preferentially interacts with its specific ligand(s). A mucin-like P-selectin glycoprotein ligand (PSGL-1) and a fibroblast growth factor-related E-selectin ligand (ESL-1) are described on endothelial cells.[17,18] Various ligands for L-selectin are described, depending on the type of endothelial cells: the sulphated glycoproteins Sgp50 (GlyCAM-1) and Sgp90 (identical to the sialomucin CD34)[19,20] and the mucosal vascular addressin MadCAM-1.[21] These ligands are mainly present on lymphoid endothelial cells. The exact inducible ligand for L-selectin on endothelial cells of postcapillary venules is until now not known. Heparin-like ligands for L-selectin are demonstrated on non-lymphoid endothelial cells,[22] supporting the predicted anionic, carbohydrate nature acting as ligand for the lectin domain of L-selectin.

The importance of carbohydrate glycans in mediating selectin-dependent leukocyte adhesion and emigration has been shown using a variety of polysaccharide structures: including (i) the inhibition of P-selectin-mediated PMN adhesion *in vitro* by dextran sulphate, heparin and fucoidan; (ii) the inhibition of leukocyte rolling in rats and rabbits by fucoidan, dextran sulphate and heparin and (iii) the reduced PMN accumulation in cerebrospinal fluid in pneumococcal antigen treated rabbits by fucoidan.[23–26]

In general PMN recruitment into tissue may be directed by different adhesion pathways depending on the type of the inflammatory agent. In the present study we have evaluated the effect of various sulphated polysaccharides on PMN extravasation and plasma leakage in response to different types of inflammatory mediators. PMN accumulation and oedema formation were measured in a rabbit skin model of acute inflammation as the local accumulation of $^{111}$In-labelled PMNs and $^{125}$I-albumin after intradermal injection of various inflammatory mediators. Different types of inflammatory agents were injected: PMN chemoattractants (FMLP, C5a desArg, $LTB_4$ and IL-8), inducing PMN extravasation and a parallel increase in vascular permeability; IL-1 evoking PMN recruitment without plasma leakage via a protein synthesis-dependent mechanism, and the permeability increasing agents histamine and thrombin generating plasma leakage not associated with PMN emigration.

### Material and Methods

### Animals

Male New Zealand White rabbits (2.5–3.5 kg body weight) were purchased from Penet Farm, Moorsel, Belgium.

### Reagents

Calcitonin gene-related peptide (CGRP), cytochalasin B (Cyt B), Evans blue dye, FMLP and 2-mercapto pyridine-N-oxide (MERC) were purchased from Sigma, Chemical Co. (St Louis, MO, USA). Thrombin (Thr) (Topostasine®) was from Hoffmann-La Roche (Basel, Switzerland). $^{111}$Indium $Cl_3$ (2 mCi in 0.2 ml sterile pyrogen free 0.04 N hydrochloric acid) and $^{125}$I-human serum albumin (50 $\mu$Ci/20 mg albumin/ml sterile pyrogen free isotonic saline) were from Amersham International (Buckinghamshire, UK). Percoll was from Pharmacia Fine Chemicals (Uppsala, Sweden). Plasmasteril (6% hydroxyethyl starch in 0.9% NaCl, sterile pyrogen free) was from Fresenius A.G. (Homburg, Germany). Sterile pyrogen-free solution of acid-citrate-dextrose (ACD) was from Baxter (Lessines, Belgium).

Sodium pentobarbitone (Nembutal®, 60 mg/ml) was from Abbott (Paris, France). Human recombinant interleukin 1$\beta$ (IL-1) was supplied by NIH (Maryland, USA) and human recombinant IL-8 was purchased from R&D Systems (Minneapolis, USA). $LTB_4$ was purchased from Cayman Chemical Co. (Ann Arbor, Michigan, USA). Histamine.2HCl was from Pharmachemic (Antwerp, Belgium). Zymosan-activated plasma (ZAP), used as a source for C5a desArg, was prepared by incubating heparinized rabbit plasma with zymosan 5 mg/ml for 30 min at 37°C. Zymosan was removed by centrifugation (3 500 rpm, 0°C). The concentration of C5a desArg in ZAP was 470 nM as determined by radioimmunoassay.[27]

R15.7 (a murine monoclonal antibody (mAb) ($IgG_1$)) directed against the common CD18-integrin) was kindly provided by Dr R. Rothlein, Boehringer Ingelheim Pharmaceuticals, Inc. (Ridgefield, Connecticut, USA). Mouse IgG, purchased from Sigma, was administered as control immunoglobulin.

Different sulphated polysaccharides were tested: dextran sulphate (mol. wt. 500 000) was from Pharmacia LKB (Uppsala, Sweden). The non-sulphated analogue dextran T500, with identical molecular weight was used as control. Heparin (porcine intestinal mucosa, grade II, 151 U/mg) was purchased from Sigma Chemical Co. (St Louis, MO, USA). Fucoidan, a fucose-4

sulphate-rich polysaccharide derived from Fucus vesiculosus, and protamine sulphate were obtained from Sigma Chemical Co. All polysaccharides were dissolved in sterile, pyrogen free salt solution (0.9%) and passed through a 0.2 $\mu$M-sterile filter before intravenous administration. All solutions were made immediately before the start of the experiment.

### Preparation of $^{111}$In-labelled PMNs

Isolation and labelling with $^{111}$In-radioisotopes of rabbit PMNs were executed as previously described.[28,29] Briefly, 72 ml of citrated blood was collected after cannulation of the carotid artery from an anaesthetized donor rabbit. PMNs were purified by a 40–60% discontinuous Percoll-plasma gradient, after initial red cell sedimentation with hydroxyethylstarch (Plasmasteril, final concentration 3%). PMNs (4–$7 \times 10^7$ cells) were incubated with $^{111}InCl_3$ (50–200 $\mu$Ci) chelated to MERC (40 $\mu$g) for 15 min at room temperature. The labelled cells were washed twice and resuspended in 6 ml autologous citrated plasma before intravenous injection into two recipient rabbits.

For *ex vivo* incubation with dextran sulphate, $^{111}$In-PMNs were divided into two equal amounts of cells (approximately $3 \times 10^7$ cells/tube) before the last wash. The cells were incubated with 5 mg of dextran sulphate or dextran T500 (as control) for 15 min in a total volume of 1 ml. Thereafter, PMNs were washed with autologous citrated plasma and injected into two recipient rabbits.

### Estimation of PMN accumulation and plasma leakage in rabbit skin

Rabbits were anaesthetized with sodium pentobarbital (30 mg/kg) and the back skin was clipped. PMN infiltration and plasma leakage were measured as the local accumulation of intravenously (i.v., −15 min) injected $^{111}$In-labelled PMNs and $^{125}$I-human serum albumin (5 $\mu$Ci mixed with 2 ml of a 2.5% Evans blue dye solution to visualize sites of plasma protein leakage). Inflammatory mediators were injected intradermally (i.d.) in 0.1 ml volumes (diluted in sterile pyrogen-free saline), each agent having six replicate injection sites per animal (according to a balanced site pattern).

Different inflammatory mediators were tested in the rabbit skin: a protein synthesis-dependent mediator: IL-1 (20–200 U/site); PMN chemoattractants: FMLP ($5.10^{-12}$–$5.10^{-11}$ mol/site), C5a desArg ($5.10^{-12}$–$5.10^{-11}$ mol/site), $LTB_4$ ($5.10^{-11}$–$5.10^{-10}$ mol/site) and IL-8 ($2.10^{-12}$–$2.10^{-11}$ mol/site); and PMN-independent, microvascular permeability increasing mediators: histamine (Hist, $10^{-8}$ mol/site) and thrombin (Thr, 1 U/site).

PMN infiltration and oedema formation in the rabbit skin are based on a synergism between an increase in local blood flow on the arteriolar side and an increase in vascular permeability on the venular side. Therefore calcitonin gene-related peptide (CGRP, $10^{-11}$ mol/site) was co-injected as vasodilator together with the inflammatory agents to increase the low basal blood flow in the back skin of the rabbit.[30]

In all *in vivo* experiments '$t = 0$' refers to the i.d. injection of various inflammatory mediators plus CGRP. IL-1 acting via a protein synthesis-dependent mechanism, was injected 3.5 h before the other inflammatory agents and before CGRP, in order to get a maximal response.[28]

After measuring PMN accumulation and plasma leakage over a period of 1 h, a blood sample was taken by cardiac puncture. Rabbits were killed by an overdose of sodium pentobarbital and the injection sites were excised with a 17 mm diameter punch. The skin samples were counted in a gamma-counter with automatic spill-over correction (Cobra 5005, Packard). PMN infiltration is expressed as the number of $^{111}$In-PMNs/site determined by comparing $^{111}$In-counts per site with $^{111}$In-counts per PMN after labelling. Plasma extravasation is expressed in terms of equivalents of $\mu$l plasma by dividing skin sample $^{125}$I-counts by $^{125}$I-counts in 1 $\mu$l plasma. Calculation of the percentage of circulating radiolabelled PMNs at the end of the experiment was based on total cell-associated activity injected and total blood volume of the recipient rabbit (7.4% expressed as percentage of the body weight).[31]

### PMN aggregation

Human PMNs were isolated from 15 ml of buffy coats (obtained from the Antwerp Transfusion Centre, Belgium) by a discontinuous Percoll-Hank's/BSA gradient, after red cell sedimentation with 3% hydroxyethylstarch. Cells were suspended in Hank's Balanced Salt Solution (1% BSA) at concentrations of $2 \times 10^7$ cells/ml. Aggregometry was performed using a Payton Dual Channel Standard platelet aggregometer, with minor modifications.[32]

Two hundred $\mu$l of the PMN suspension was added to a siliconized cuvette with a Teflon stirring bar revolving at 600 rpm. $Ca^{2+}/Mg^{2+}$ (0.5 mM as a final concentration) and cytochalasin B (Cyt B, 10 $\mu$g/ml) was added to the cells after warming up to 37°C during 1 min. After addition of FMLP ($10^{-6}$ M) the resulting changes in light transmission were recorded in function of time. Test agents were added 1 min prior to the addition of $Ca^{2+}/Mg^{2+}$. All working solutions were made in Hank's Balanced Salt Solution with 1% BSA.

### Statistics

All data are reported as the mean ± standard error of the mean (SEM) for the indicated number of experiments and have been analysed using a Student's *t*-test for unpaired investigations. Statistical significance was set at $p < 0.05$.

### Results

### Effect of dextran sulphate, heparin or fucoidan on PMN infiltration and plasma leakage

In control-treated animals (dextran T500, 25 mg/kg), PMN accumulation and plasma leakage dose-dependently increased after local injection of the PMN chemoattractants FMLP, C5a desArg, $LTB_4$ and IL-8 (Fig. 1). PMN infiltration induced by IL-1 was not associated with detectable oedema formation. Histamine and thrombin evoked an increase in microvascular plasma leakage without a significant increase in PMN accumulation. Intravenous injection of dextran sulphate (25 mg/kg, 30 min before the start of the experiment), completely abolished PMN infiltration and plasma extravasation in response to the PMN chemoattractants. This concentration of dextran sulphate had been shown to inhibit leukocyte rolling in rabbit mesenteric venules for more than 90%.[24] In contrast, PMN accumulation in response to IL-1 was not suppressed by dextran sulphate and it did not affect the PMN-independent increase in vascular permeability induced by histamine and thrombin. The inhibition of PMN extravasation by dextran sulphate was not due to an effect on the circulating number of $^{111}$In-labelled PMNs measured at the end of the experiments (dextran sulphate: 51 ± 3% *vs* dextran T500: 49 ± 3%).

IL-1 was injected i.d. 3.5 h before the other inflammatory mediators and before CGRP ($10^{-11}$ mol/site), but also 3 h before the intravenous injection of dextran sulphate. To exclude the possibility that a time-related phenomenon could be responsible for the lack of effect of dextran sulphate on the IL-1-induced PMN infiltration, dextran sulphate was also injected just before the i.d. injection of IL-1 ($t = -3.5$ h). Although the polysaccharide still reduced the FMLP-induced PMN infiltration and plasma leakage 3.5 h later, it did not affect the IL-1 mediated responses (Fig. 2).

Interference of dextran sulphate at the level of chemoattractant/receptor interaction is unlikely, since local administration of dextran sulphate (1–100 μg/site) together with FMLP or histamine did not inhibit plasma leakage (data not shown).

The direct effect of dextran sulphate on PMNs was also investigated. In these experiments, $^{111}$In-labelled PMNs were incubated *ex vivo* with dextran sulphate (5 mg) or dextran T500 (control) before i.v. injection of the PMNs. As shown in Table 1, *ex vivo* incubation of $^{111}$In-PMNs with dextran sulphate did not suppress PMN infiltration. The lack of effect of dextran sulphate on PMN extravasation after *ex vivo* incubation may correspond with the reversible binding of dextran sulphate to PMNs.[33]

In comparison to dextran sulphate a higher dose of heparin was needed to inhibit leukocyte rolling.[34] The dose of heparin (90 mg/kg), which was required to inhibit leukocyte rolling for >90%, was not sufficient to significantly suppress PMN infiltration in the rabbit skin measured over a period of 1 h (data not shown). Therefore two bolus injections of heparin (90 mg/kg) were injected i.v. (15 min before and 15 min after i.d. injection of the inflammatory agents). As shown in Fig. 3, this dose of heparin resulted in a significant but incomplete inhibition of PMN infiltration and plasma leakage induced by the PMN chemoattractants. PMN recruitment in response to IL-1 was not significantly suppressed. Again plasma leakage induced by histamine and thrombin was not affected.

The ligand activity of fucoidan for L-selectin was described to be at least 100-fold more potent per unit weight than heparin.[35] Therefore, 1 mg/kg fucoidan was injected 15 min before the start of the experiment. This dose of fucoidan significantly inhibited PMN infiltration for 60–70% (after background substraction) and oedema formation for 50–60% in response to the chemoattractants (Fig. 4). In contrast to the results obtained after administration of dextran sulphate or heparin, fucoidan also reduced PMN recruitment after IL-1 injection. Fucoidan did not affect the percentage of circulating $^{111}$In-PMNs as measured at the end of the experiment (fucoidan: 48 ± 8% *vs* control: 54 ± 8%).

Protamine sulphate (i.v., 25 mg/kg), also shown to inhibit leukocyte rolling in mesenteric postcapillary venules,[34] could not be administered in this model since systemic administra-

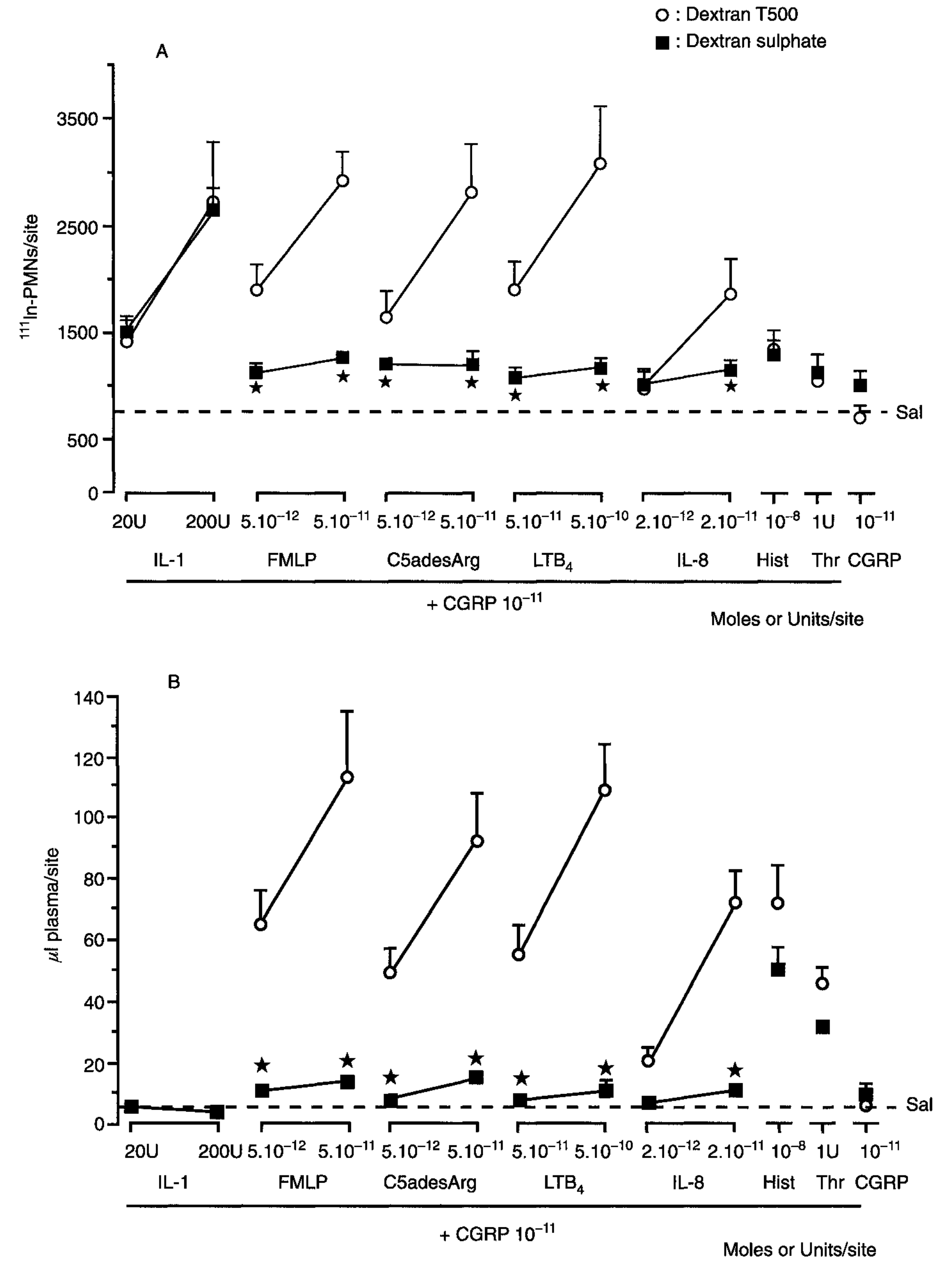

FIG. 1. Effect of dextran sulphate (25 mg/kg, −30 min, ■) *vs* dextran T500 (25 mg/kg, O) on PMN recruitment (expressed as $^{111}$In-PMNs/site) (panel A) and plasma albumin leakage (expressed as μl plasma/site) (panel B). IL-1, FMLP, C5a desArg, LTB$_4$, IL-8, Hist and Thr were injected i.d. in the presence of CGRP ($10^{-11}$ mol/site). The dashed line represents the response to i.d. injection of saline. The results are expressed as the mean ± SEM for $n = 7$ rabbits (* $p < 0.01$, Student's *t*-test: dextran sulphate *vs* dextran T500).

tion resulted in a dramatic decrease of the mean arterial blood pressure in the rabbit (data not shown).

### Estimation of CD18-mediated PMN infiltration in rabbit skin

Anti-CD18 (R15.7, 2 mg/kg) or mouse IgG (2 mg/kg) were injected i.v. 30 min before the start of the experiment (Fig. 5). PMN infiltration and plasma leakage in response to the PMN chemoattractants C5a desArg, FMLP, LTB$_4$ and IL-8 were completely suppressed by anti-CD18. IL-1-induced PMN accumulation was suppressed for 60–70% in anti-CD18 treated animals. Plasma leakage induced by thrombin was not affected.

### Effect of dextran sulphate on FMLP-induced PMN aggregation

PMNs were incubated with dextran sulphate or dextran T500 (final concentration 300 μg/ml) during 1 min at 37°C, under stirring conditions. This *in vitro* concentration is comparable with the *in vivo* injected dose of 25 mg/kg, taken

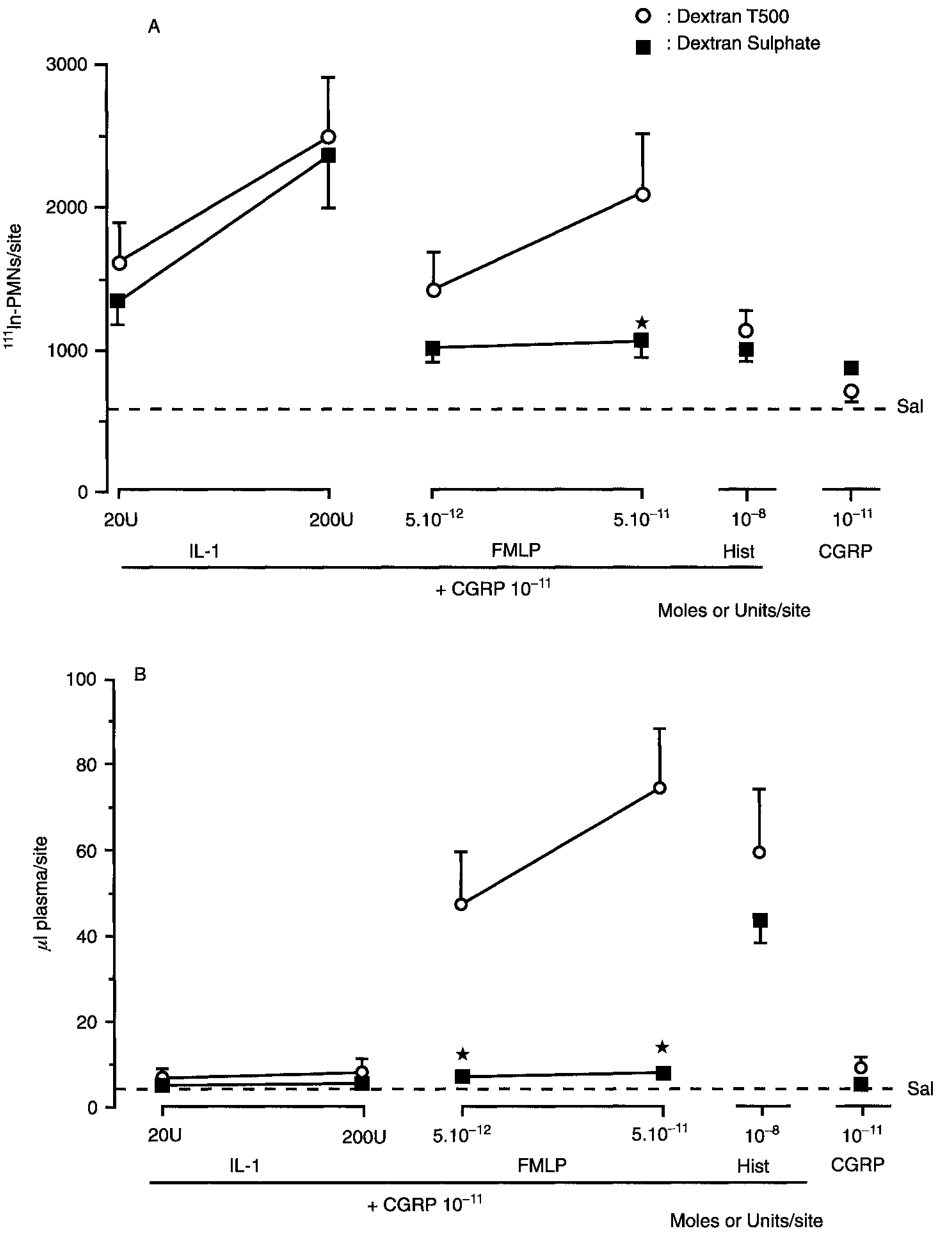

FIG. 2. PMN infiltration ($^{111}$In-PMNs/site, panel A) and oedema formation ($\mu$l plasma/site, panel B) measured in rabbits after systemic administration of dextran sulphate (25 mg/kg, ■) or dextran T500 (25 mg/kg, O) 3.5 h prior to the start of the experiment. Results are expressed as the mean ± SEM for $n = 4$ rabbits ($^*p < 0.01$, Student's *t*-test: dextran sulphate *vs* dextran T500).

into account that the blood volume in the rabbit is about 7.4% of its body weight.[31] After recalcification ($Ca^{2+}/Mg^{2+}$, 0.5 mM) and addition of Cyt B (10 $\mu$g/ml), PMNs were stimulated with FMLP ($10^{-6}$ M). As shown in Fig. 6, PMN aggregation in the presence of dextran T500 (300 $\mu$g/ml) was not altered as compared to control aggregation. Although the maximal aggregation was not altered in the presence of dextran sulphate, the aggregation was delayed. Similar results were obtained with heparin (data not shown). In contrast, fucoidan (20 $\mu$g/ml) had no effect on PMN aggregation. Under the same experimental conditions, anti-CD18 suppressed PMN aggregation by more than 70% (R 15.7 10 $\mu$g/ml, *vs* mouse IgG).

### Discussion

Rolling of leukocytes along endothelial cells is mediated by various selectins: L-selectin on leukocytes and P- and E-selectin on activated endothelial cells. All three selectins are recognizing carbohydrate-based ligands on opposing cells via their $Ca^{2+}$-dependent lectin domain, with a common recognition motif sialyl Lewis x ($SLe^x$).[36] Sulphated polysaccharides, like dextran sulphate and fucoidan can disturb selectin-

**Table 1.** Effect of *ex vivo* incubation of $^{111}$In-PMNs with dextran sulphate (5 mg) or dextran T500 on PMN accumulation (expressed as $^{111}$In-PMNs/site) *in vivo*

| Mediator | Concentration | $^{111}$In-PMNs/site | |
|---|---|---|---|
| | | Dextran T500 | Dextran sulphate |
| IL-1 | 200 U/site | 2459 ± 193 | 2172 ± 441 |
| FMLP | 5 × $10^{-11}$ mol/site | 3378 ± 216 | 3010 ± 756 |
| C5a desArg | 5 × $10^{-11}$ mol/site | 3029 ± 274 | 2613 ± 411 |
| CGRP | $10^{-11}$ mol/site | 611 ± 66 | 540 ± 11 |

The results are expressed as the mean ± SEM for $n = 5$ rabbits. All mediators were tested in the presence of CGRP ($10^{-11}$ mol/site) as vasodilator.

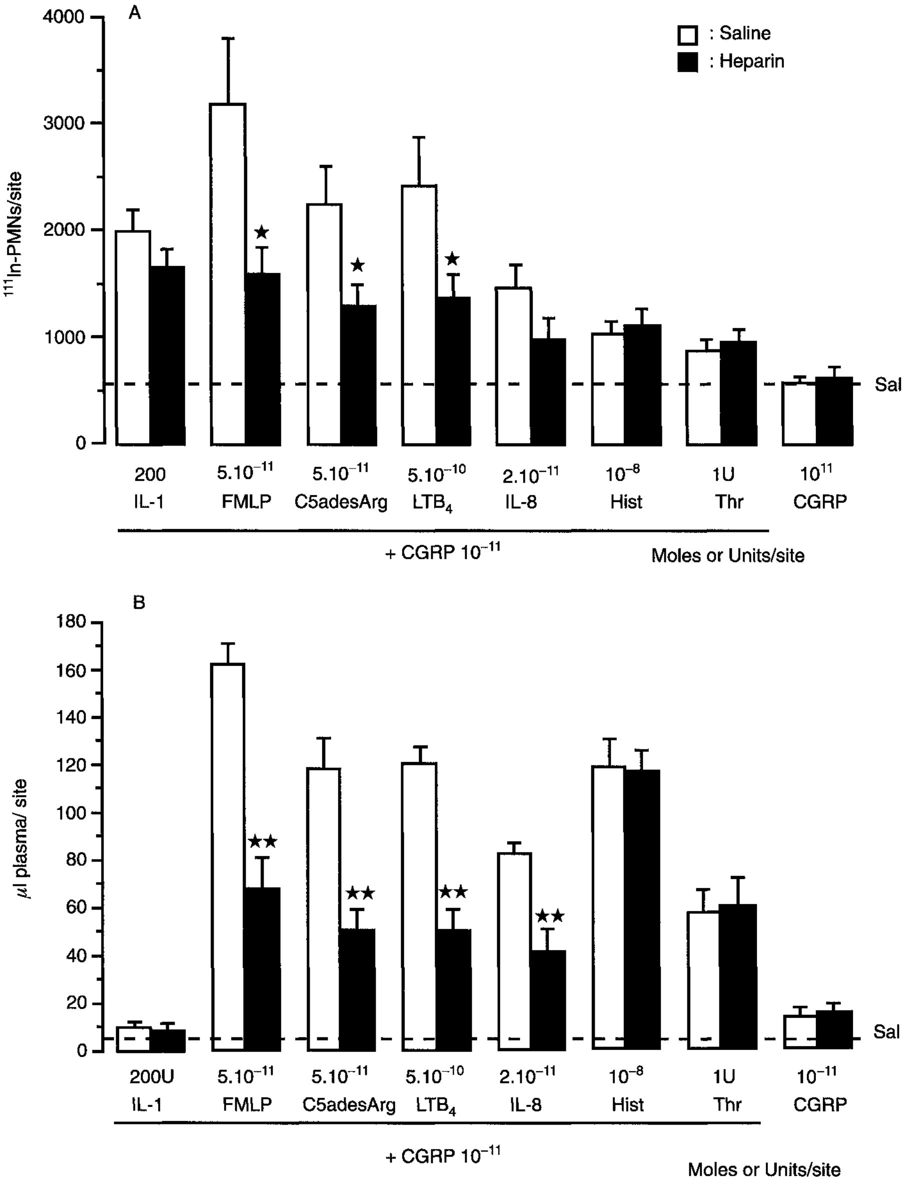

FIG. 3. Effect of two bolus injections of heparin (90 mg/kg, 15 min before and 15 min after i.d. injection of PMN chemoattractants plus CGRP) (closed bars) *vs* saline (open bars) on PMN infiltration (panel A) and plasma leakage (panel B). Results are expressed as the mean ± SEM for $n = 7$ rabbits (* $p < 0.05$, Student's *t*-test: heparin *vs* saline).

mediated rolling of PMNs along the endothelial cells in postcapillary venules.[24,25] In our rabbit skin model of acute inflammation dextran sulphate selectively inhibited PMN infiltration and associated plasma leakage in response to intradermal injection of various PMN chemoattractants (FMLP, C5a desArg, IL-8 and $LTB_4$), without suppressing IL-1-induced PMN infiltration.

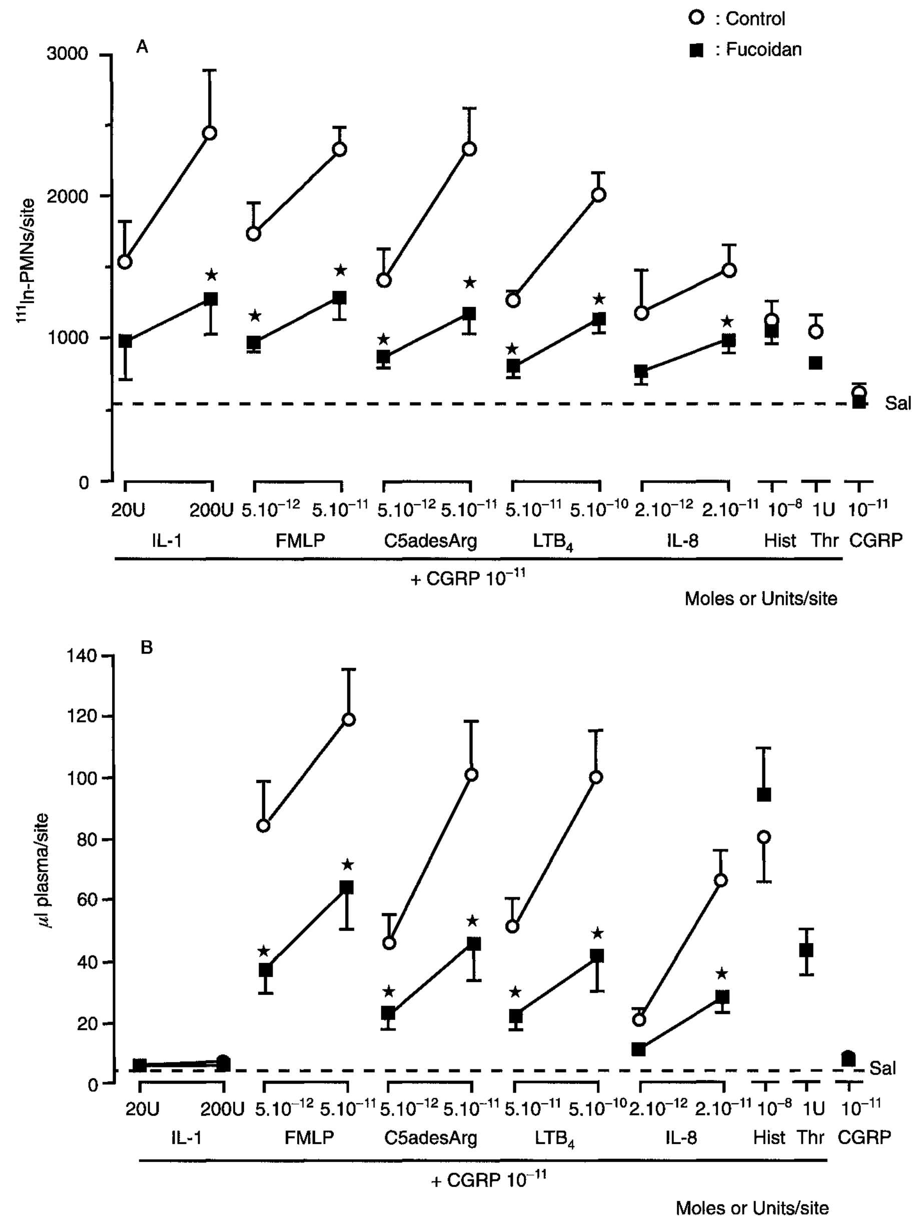

FIG. 4. PMN infiltration (panel A) and oedema formation (panel B) in response to different inflammatory mediators, after i.v. injection of fucoidan (1 mg/kg, −15 min, ■) *vs* control (saline, 1 ml/kg, O). Results are expressed as the mean ± SEM for $n = 5$–7 rabbits (* $p < 0.05$, Student's *t*-test: fucoidan *vs* saline).

Since the increase in vascular permeability in response to histamine and thrombin was not affected by dextran sulphate, we can exclude possible dextran sulphate-induced changes in local blood flow. The complete inhibition of PMN infiltration by dextran sulphate was comparable with the more than 90% reduction of leukocyte rolling in rabbit mesentery venules, obtained by Ley *et al.*[24] The presence of sulphate on glycans is crucial since other negatively charged or neutral polysaccharides had no effect on leukocyte rolling.[34]

The possible involvement of sulphated glycoconjugates during leukocyte-endothelial cell interaction is demonstrated at different levels. The interaction of sulphated and phosphated polysaccharides (i.e. dextran sulphate, fucoidan and PPME (a yeast polysaccharide of mannose and mannose-6-phosphate) with L-selectin has been demonstrated *in vitro* and suggested *in vivo*.[37–39] Rapid sulphate uptake by high endothelial cells in the venous microvasculature is correlated with increased lymphocyte accumulation, most likely mediated by the homing receptor L-selectin.[8,40] An internal pool of heparin-like chains, recognizing L-selectin was found in cultured venular endothelial cells.[22] The sulphatation of the endothelial cell ligand Gly-CAM-1 was demonstrated to be essential for the binding to L-selectin.[41] The sulphated form

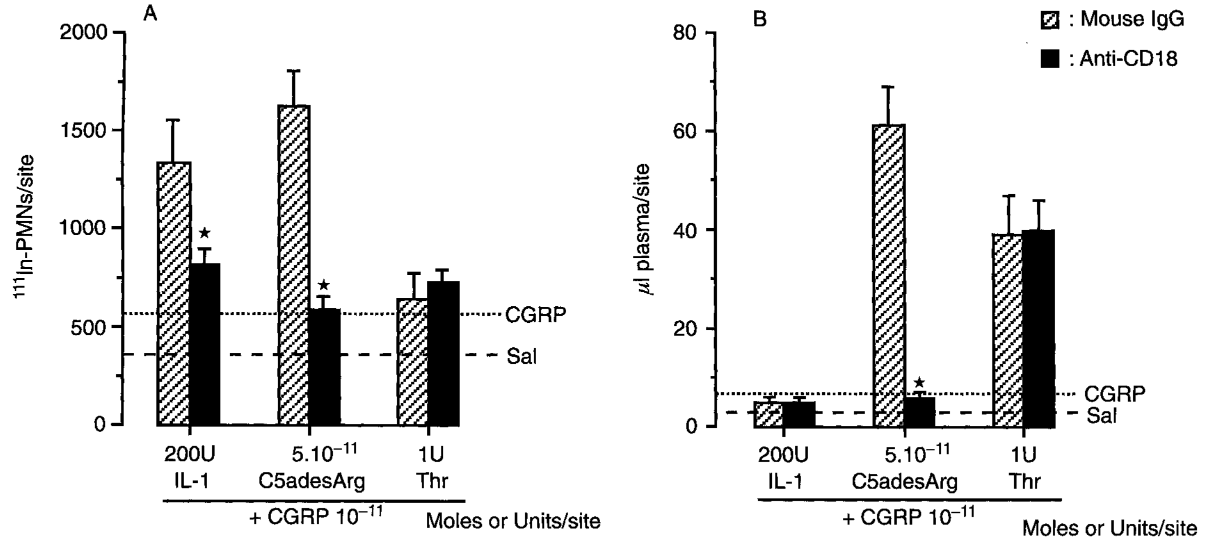

FIG. 5. Inhibition of PMN infiltration (panel A) and plasma leakage (panel B) in rabbit skin after administration of anti-CD18 (2 mg/kg, closed bars) *vs* mouse IgG as control (2 mg/kg, hatched bars). PMN infiltration and plasma leakage in response to i.d. injection of IL-1, C5a desArg and thrombin are shown. Similar results as for C5a desArg were obtained after i.d. injection of the other PMN chemoattractants (FMLP, $LTB_4$ and IL-8). Results are expressed as the mean ± SEM for $n = 4$ rabbits (* $p < 0.05$, Student's *t*-test: anti-CD18 *vs* mouse IgG).

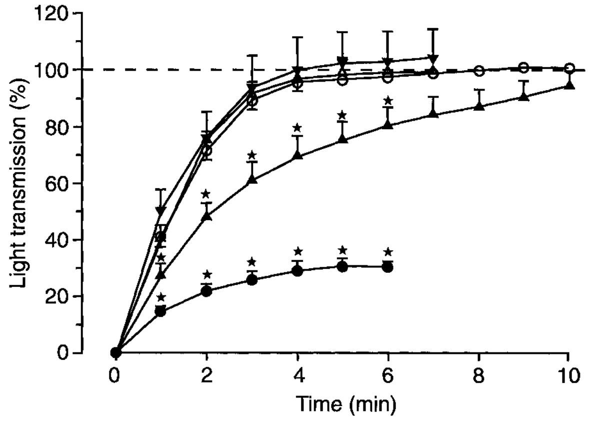

FIG. 6. Effect of dextran sulphate (300 μg/ml, ▲) or dextran T500 (300 μg/ml, △) and fucoidan (20 μg/ml, ▼, on PMN aggregation, measured as percentage change in light transmission *vs* control (○). Anti-CD18 (R15.7, 10 μg/ml) (●) was added to investigate CD18-dependent aggregation. Results are expressed as the mean percentage change in light transmission ± SEM ($n = 5$–$7$) (* $p < 0.05$, Student's *t*-test: dextran sulphate *vs* dextran T500).

of SLe$^x$ (6′-sulphated SLe$^x$), capping the GlyCAM-1 structure, binds to L-selectin with higher affinity than SLe$^x$.[42] Tyrosine sulphatation of the P-selectin glycoprotein ligand-1 (PSGL-1) structure is in addition to the appearance of fucosylated, sialylated glycans important for the binding of P-selectin but of no importance for the recognition of E-selectin.[43]

Inhibition of PMN infiltration after administration of heparin was similar to the effect of dextran sulphate, although less pronounced. The lower potency of heparin to reduce PMN extravasation is consistent with its lower potency to block leukocyte rolling.[34] Administration of high doses of heparin as anti-inflammatory agent may be irrelevant, because of its strong anticoagulant activity. Recently, heparin oligosaccharides without significant anticoagulant activity have been demonstrated to inhibit P- and L-selectin-dependent PMN infiltration in a murine model of thioglycollate-induced peritoneal inflammation.[38]

In contrast to dextran sulphate, fucoidan (1 mg/kg) not only suppressed PMN and plasma protein accumulation in response to PMN chemoattractants but also attenuated IL-1-induced PMN recruitment for about 60–70%. It is important to know that fucoidan had no effect on the number of circulating PMNs. Similar results were obtained by Lindbom *et al.* in rat mesenteric microvessels using intravital microscopy.[44] They achieved a 70% inhibition of leukocyte adhesion, via a rolling-dependent, CD11/CD18-independent mechanism after administration of 1 mg/kg of fucoidan. Fucoidan has been demonstrated to attenuate leukocyte- and plasma protein extravasation into cerebrospinal fluid of rabbits challenged with pneumococcal antigen.[26]

Our *in vivo* results have shown a different inhibitory pattern of fucoidan and dextran sulphate for IL-1-induced PMN accumulation. The exact mechanism is until now not known. Different adhesion pathways or different binding sites may be the reason for a complete inhibitory effect by fucoidan and only a partial reduction after administration of dextran sulphate. IL-1-activated venular endothelium may express a ligand for L-selectin with binding characteristics different from that expressed after administration of PMN chemoattractants. This ligand may be E-selectin, as suggested by Kishimoto *et al.*,[45] or a new until now not well defined, inducible ligand as demonstrated by Spertini *et al.*[46] Differences in binding to L-selectin have been described for PPME and fucoidan.[37] Both PPME and fucoidan are able to block L-selectin-mediated murine lymphocyte and PMN adhesion to lymph node high endothelial venules (HEV) *in vitro*, but only fucoidan is capable to inhibit L-selectin-mediated leukocyte rolling in venules of rat mesentery.[25] Nevertheless identical binding characteristics to P- and L-selectin were described for fucoidan and dextran sulphate, whereas no binding was found to E-selectin.[47,48] In contrast to identical binding characteristics, functional differences between fucoidan and dextran sulphate were found after *in vitro* incubation of PMNs followed by stimulation. The rate and extent of formyl-peptide-induced L-selectin shedding is consistently reduced after treatment of PMNs with fucoidan as compared with dextran sulphate-treated PMNs.[49]

It is unlikely that the sulphated polysaccharides are acting on the level of the PMN $\beta_2$-integrins (CD11/CD18). As demonstrated by Ley *et al.*, dextran sulphate up to concentrations of 1 mg/ml did not alter CD11b/CD18 expression.[33] Furthermore, both fucoidan and dextran sulphate failed to inhibit CD11b/CD18-dependent PMN adhesion under stationary conditions *in vitro*.[33,44] In addition, selectin-mediated rolling of leukocyte is blocked by sulphated polysaccharides, whereas anti-CD18 antibodies had no effect.[15] In our model dextran sulphate did not inhibit IL-1-induced PMN accumulation, whereas monoclonal antibodies against CD18 reduced PMN accumulation in response to IL-1 in this rabbit skin model. A non-specific increase in charge density on the PMN-surface by dextran sulphate is unlikely to be the cause of the inhibition, because a similar inhibitory effect of dextran sulphate would then be expected after IL-1 injection. CD18-dependent PMN aggregation induced by FMLP was delayed in the presence of dextran sulphate, however not suppressed. In contrast, fucoidan had no attenuating effect on PMN aggregation. Our results are in agreement with the observations of Bazzoni *et al.*, who found a reduced PMN aggregation in the presence of heparin measured at 3 min after stimulation.[50] The delay in aggregation may be due to sterical hindrance of dextran sulphate at the PMN surface or due to an interference on the lectin-dependent PMN aggregation.

All three selectins are possible candidates to interact with the sulphated polysaccharides. Differential involvement of P- and E-selectin in addition to L-selectin may be important in PMN recruitment in rabbit skin after injection of PMN chemoattractants or IL-1. E-selectin mediates leukocyte rolling in IL-1-treated rabbit mesentery venules.[15] A cytokine-inducible ligand for L-selectin is described on nonlymphoid cells.[22] P-selectin is translocated within minutes to the cell surface after stimulation with thrombin, histamine or oxygen radicals.[10] Recently a cytokine upregulation of P-selectin with a time course similar to that of E-selectin expression was also found.[51,52]

Participation of P-selectin in PMN infiltration in response to the different inflammatory mediators in this skin model is still a question mark. The sulphated polysaccharides, like dextran sulphate, heparin and fucoidan are shown to disrupt P-selectin-mediated PMN interaction *in vitro*.[23,53] Until now, there is no direct evidence to suggest that P-selectin is expressed on endothelial cells after stimulation with pure PMN chemoattractants such as FMLP or IL-8. Under those conditions histamine is not released as secondary mediator in response to IL-1 or PMN chemoattractants.[54] In our model the sulphated polysaccharides had no effect on histamine- or thrombin-related increase in microvascular permeability. Nevertheless, neuropeptides such as calcitonin gene-related peptide (CGRP), locally injected in normal human skin, translocate P-selectin within 15 min to the luminal membrane.[55] Since in our model CGRP is co-injected to enhance the local blood flow, the role of P-selectin during PMN infiltration can therefore not be excluded.

The relative role of the selectins may depend on the particular mediators and on the inflammatory model being studied. The selectin-mediated adhesion pathways of leukocytes on endothelial cells are partially redundant, partially separate.[56,57] The combined involvement of different selectins was demonstrated *in vivo* by Mulligan in two models of lung inflammation using selectin antibodies. Acute lung injury induced by Cobra Venom Factor, measured after 30 min, was mediated by both L- and P-selectin, whereas IgG immune complex-induced injury, measured after a period of 4 h was L- and E-selectin-dependent.[58] Recent studies on animals genetically deficient in these selectin have shed light on the temporal sequence of selectin functions, as well as on their individual contributions in acute inflammatory models.[59] Early after injury leukocyte rolling was defective and PMN emigration was delayed in P-selectin knock-out mice, whereas later on L-selectin-mediated rolling appeared to be more prominent.[56,60] Discrimination between the different selectins can also be made on the level of oedema formation. In a model of contact-hypersensitivity plasma leakage was increased via a L-selectin-dependent mechanism with only a minor role for P-selectin.[61,62] Which adhesion pathways are involved during PMN recruitment evoked by the chemoattractants and by IL-1 in the rabbit skin is at present not clear. Different infiltration pathways in response to IL-1 or to the chemoattractant FMLP were also demonstrated by Wakelin *et al.*[63] With mAb against PECAM-1 IL-1-induced PMN extravasation across rat mesenteric microvessels could be suppressed, whereas anti-PECAM-1 had no effect on FMLP-evoked PMN infiltration. There is, however, no evidence that dextran sulphate or fucoidan can interfere with the PECAM-1 adhesion pathway.

The exact mechanism behind the inhibition of PMN infiltration and oedema formation by the sulphated polysaccharides is not clear. It cannot be excluded that dextran sulphate and heparin might interfere with PMN-derived polycations, such as elastase, cathepsin G or defensins to reduce PMN recruitment.[64,65] However, the direct inhibition of elastase by dextran sulphate is unlikely, since $LTB_4$-induced PMN-dependent increase of plasma leakage in the hamster cheek pouches was significantly suppressed by dextran sulphate, whereas administration of elastase inhibitors had no effect.[65] The role of other naturally occurring cationic peptides, derived from PMNs, during the induction of plasma leakage cannot, however, be excluded.

Nevertheless, since the recent selectin-carbohydrate binding studies, there is increasing and attractive evidence to suggest that the anti-infiltrating properties of these sulphated polysaccharides are attributed to their interference with the selectins.[25,38] Although the different selectin adhesion molecules can commonly bind to $SLe^x$ and related compounds, the knowledge of the specific binding characteristics of the carbohydrate structure with each selectin, can contribute to a more efficient anti-adhesion therapy.

We have demonstrated that sulphated glycans may be involved during PMN infiltration and plasma leakage in the rabbit skin unrelated to the CD11b/CD18 adhesion pathway. Since dextran sulphate and fucoidan differently inhibit PMN infiltration *in vivo*, depending on the type of inflammatory stimulus, different sulphated glycans may be involved during PMN recruitment.

### References

1. von Andrian UH, Chambers JD, McEvoy LM, Bargatze RF, Arfors KE, Butcher EC. Two-step model of leukocyte-endothelial cell interaction in inflammation: distinct roles for LECAM-1 and the leukocyte $\beta 2$ integrins *in vivo*. *Proc Natl Acad Sci USA* 1991; **88**: 7538–7542.
2. Butcher EC. Leukocyte-endothelial cell recognition: three (or more) steps to specificity and diversity. *Cell* 1991; **67**: 1033–1036.
3. Tedder TF, Steeber DA, Chen A, Engel P. The selectins: vascular adhesion molecules. *FASEB J* 1995; **9**: 866–873.
4. McEver RP, Moore KL, Cummings RD. Leukocyte trafficking mediated by selectin-carbohydrate interactions. *J Biol Chem* 1995; **270**: 11025–11028.
5. Arfors KE, Lundberg C, Lindbom L, Lundberg K, Beatty PG, Harlan JM. A monoclonal antibody to the membrane glycoprotein complex CD18 inhibits polymorphonuclear leukocyte accumulation and plasma leakage *in vivo*. *Blood* 1987; **69**: 338–340.
6. Argenbright LW, Letts LG, Rothlein R. Monoclonal antibodies to the leukocyte membrane CD18 glycoprotein complex and to intercellular adhesion molecule-1 inhibit leukocyte-endothelial adhesion in rabbits. *J Leukoc Biol* 1991; **49**: 253–257.
7. Vaporciyan AA, DeLisser HM, Yan H-C, *et al.* Involvement of platelet-endothelial cell adhesion molecule-1 in neutrophil recruitment *in vivo*. *Science* 1993; **262**: 1580–1582.
8. Gallatin WM, Weissman IL, Butcher EC. A cell-surface molecule involved in organ-specific homing of lymphocytes. *Nature* 1983; **304**: 30–34.
9. Spertini O, Kansas GS, Munro JM, Griffin JD, Tedder TF. Regulation of leukocyte migration by activation of the leukocyte adhesion molecule-1 (LAM-1) selectin. *Nature* 1991; **349**: 691–694.
10. McEver RP, Beckstead JH, Moore KL, Marshall-Carlson L, Bainton DF. GMP-140, a platelet $\alpha$-granule membrane protein, is also synthesized by vascular endothelial cells and is localized in Weibel-Palade bodies. *J Clin Invest* 1989; **84**: 92–99.
11. Bevilacqua MP, Stengelin S, Gimbrone MA, Jr, Seed B. Endothelial leukocyte adhesion molecule 1: an inducible receptor for neutrophils related to complement regulatory proteins and lectins. *Science* 1989; **243**: 1160–1165.
12. Doré M, Korthuis RJ, Granger DN, Entman ML, Smith CW. P-selectin mediates spontaneous leukocyte rolling *in vivo*. *Blood* 1993; **82**: 1308–1316.
13. Abbassi O, Kishimoto TK, McIntire LV, Anderson DC, Smith CW. E-selectin supports neutrophil rolling *in vitro* under conditions of flow. *J Clin Invest* 1993; **92**: 2719–2730.
14. Watson SR, Fennie C, Lasky LA. Neutrophil influx into an inflammatory site inhibited by a soluble homing receptor-IgG chimaera. *Nature* 1991; **349**: 164–167.
15. Olofsson AM, Arfors K-E, Ramezani L, Wolitzky BA, Butcher EC, von Andrian UH. E-selectin mediates leukocyte rolling in interleukin-1-treated rabbit mesentery venules. *Blood* 1994; **84**: 2749–2758.
16. Varki A. Selectin ligands. *Proc Natl Acad Sci USA* 1994; **91**: 7390–7397.
17. Moore KL, Stults NL, Diaz S, *et al.* Identification of a specific glycoprotein ligand for P-selectin (CD62) on myeloid cells. *J Cell Biol* 1992; **118**: 445–456.
18. Steegmaler M, Levinovitz A, Isenmann S, *et al.* The E-selectin-ligand ESL-1 is a variant of a receptor for fibroblast growth factor. *Nature* 1995; **373**: 615–620.
19. Lasky LA, Singer MS, Dowbenko D, *et al.* An endothelial ligand for L-selectin is a novel mucin-like molecule. *Cell* 1992; **69**: 927–938.
20. Baumhueter S, Singer MS, Henzel W, *et al.* Binding of L-selectin to the vascular sialomucin CD34. *Science* 1993; **262**: 436–438.
21. Berg EL, McEvoy LM, Berlin C, Bargatze RF, Butcher EC. L-selectin-mediated lymphocyte rolling on MAdCAM-1. *Nature* 1993; **366**: 695–698.
22. Norgard-Sumnicht KE, Varki NM, Varki A. Calcium-dependent heparin-like ligands for L-selectin in nonlymphoid endothelial cells. *Science* 1993; **261**: 480–483.
23. Skinner MP, Lucas CM, Burns GF, Chesterman CN, Berndt MC. GMP-140 binding to neutrophil is inhibited by sulfated glycans. *J Biol Chem* 1991; **266**: 5371–5374.
24. Ley K, Cerrito M, Arfors KE. Sulfated polysaccharides inhibit leukocyte rolling in rabbit mesentery venules. *Am J Physiol* 1991; **260**: 1667H–1673H.
25. Ley K, Linneman G, Meinen M, Stoolman LM, Gaehtgens P. Fucoidin, but not yeast polyphosphomannan PPME, inhibits leukocyte rolling in venules of the rat mesentery. *Blood* 1993; **81**: 177–185.
26. Granert C, Raud J, Xie C, Lindquist L, Lindbom L. Inhibition of leukocyte rolling with polysaccharide fucoidin prevents pleocytosis in experimental meningitis in the rabbit. *J Clin Invest* 1994; **93**: 929–936.

27. Jose PJ, Forrest MJ, Williams TJ. Detection of the complement fragment C5a in inflammatory exudates from the rabbit peritoneal cavity using radioimmunoasay. *J Exp Med* 1983; **158**: 2177–2182.
28. Rampart M, Williams TJ. Evidence that neutrophil accumulation induced by interleukin-1 requires both local protein biosynthesis and neutrophil CD18 antigen expression *in vivo*. *Br J Pharmacol* 1988; **94**: 1143–1148.
29. Van Osselaer N, Van Damme J, Rampart M, Herman AG. Increased microvascular permeability *in vivo* in response to intradermal injection of neutrophil-activating protein (NAP-2) in rabbit skin. *Am J Pathol* 1991; **138**: 23–27.
30. Brain SD, Williams TJ. Inflammatory oedema induced by synergism between calcitonin gene-related peptide (CGRP) and mediators of increased vascular permeability. *Br J Pharmacol* 1985; **86**: 855–860.
31. Nourshargh S, Williams TJ. Evidence that a receptor-operated event on the neutrophil mediates neutrophil accumulation *in vivo*. Pretreatment of $^{111}$In-neutrophils with pertussis toxin *in vitro* inhibits their accumulation *in vivo*. *J Immunol* 1990; **145**: 2633–2638.
32. Craddock PR, Hammerschmidt D, White JG, Dalmasso AP, Jacob HS. Complement (C5a)-induced granulocyte aggregation *in vitro*. A possible mechanism of complement-mediated leukostasis and leukopenia. *J Clin Invest* 1977; **60**: 260–264.
33. Ley K, Lundgren E, Berger E, Arfors KE. Shear-dependent inhibition of granulocyte adhesion to cultured endothelium by dextran sulfate. *Blood* 1989; **73**: 1324–1330.
34. Tangelder GJ, Arfors KE. Inhibition of leukocyte rolling in venules by protamine and sulfated polysaccharides. *Blood* 1991; **77**: 1565–1571.
35. Imai Y, True DD, Singer MS, Rosen SD. Direct demonstration of the lectin activity of gp90MEL, a lymphocyte homing receptor. *J Cell Biol* 1990; **111**: 1225–1232.
36. Foxall C, Watson SR, Dowbenko D, *et al.* The three members of the selectin receptor family recognize a common carbohydrate epitope, the sialyl Lewis X oligosaccharide. *J Cell Biol* 1992; **117**: 895–902.
37. Yednock TA, Butcher EC, Stoolman LM, Rosen SD. Receptors involved in lymphocyte homing: relationship between a carbohydrate-binding receptor and the MEL-14 antigen. *J Cell Biol* 1987; **104**: 725–731.
38. Nelson RM, Cecconi O, Roberts WG, Aruffo A, Linhardt RJ, Bevilacqua MP. Heparin oligosaccharides bind L- and P-selectin and inhibit acute inflammation. *Blood* 1993; **82**: 3253–3258.
39. Stoolman LM, Rosen SD. Possible role for cell-surface carbohydrate-binding molecules in lymphocyte recirculation. *J Cell Biol* 1983; **96**: 722–729.
40. Freemont AJ. Functional and biosynthetic changes in endothelial cells of vessels in chronically inflamed tissues: evidence for endothelial control of lymphocyte entry into diseased tissues. *J Pathol* 1988; **155**: 225–230.
41. Imai Y, Lasky LA, Rosen SD. Sulphation requirement for GlyCAM-1, an endothelial ligand for L-selectin. *Nature* 1993; **361**: 555–557.
42. Hemmerich S, Rosen SD. 6′-sulfated sialyl Lewis x is a major capping group of GlyCAM-1. *Biochemistry* 1994; **33**: 4830–4835.
43. Pouyani T, Seed B. PSGL-1 recognition of P-selectin is controlled by a tyrosine sulfation consensus at the PSGL-1 amino terminus. *Cell* 1995; **83**: 333–343.
44. Lindbom L, Xie X, Raud J, Hedqvist P. Chemoattractant-induced firm adhesion of leukocytes to vascular endothelium *in vivo* is critically dependent on initial leukocyte rolling. *Acta Physiol Scand* 1992; **146**: 415–421.
45. Kishimoto TK, Warnock RA, Jutila MA, *et al.* Antibodies against human neutrophil LECAM-1 (LAM-1/Leu-8/DREG-56 antigen) and endothelial cell ELAM-1 inhibit a common CD18-independent adhesion pathway *in vitro*. *Blood* 1991; **78**: 805–811.
46. Spertini O, Luscinskas FW, Kansas GS, *et al.* Leukocyte adhesion molecule-1 (LAM-1, L-selectin) interacts with an inducible endothelial cell ligand to support leukocyte adhesion. *J Immunol* 1991; **147**: 2565–2573.
47. Tyrell D, James P, Rao N, *et al.* Structural requirements for the carbohydrate ligand of E-selectin. *Proc Natl Acad Sci USA* 1991; **88**: 10372–10376.
48. Nelson RM, Dolich S, Aruffo A, Cecconi O, Bevilacqua MP. Higher-affinity oligosaccharide ligands for E-selectin. *J Clin Invest* 1993; **91**: 1157–1166.
49. Rochon YP, Simon SI, Lynam EB, Sklar LA. A role for lectin interactions during human neutrophil aggregation. *J Immunol* 1994; **152**: 1385–1393.
50. Bazzoni G, Nunez AB, Mascellani G, Bianchini P, Dejana E, Del Maschio A. Effect of heparin, dermatan sulfate, and related oligo-derivatives on human polymorphonuclear leukocyte functions. *J Lab Clin Med* 1993; **121**: 268–275.
51. Hahne M, Jäger U, Isenmann S, Hallmann R, Vestweber D. Five tumor necrosis factor-inducible cell adhesion mechanisms on the surface of mouse endothelioma cells mediate the binding of leukocytes. *J Cell Biol* 1993; **121**: 655–664.
52. Weller A, Isenmann S, Vestweber D. Cloning of the mouse endothelial selectins. Expression of both E- and P-selectin is inducible by tumor necrosis factor $\alpha$. *J Biol Chem* 1992; **267**: 15176–15183.
53. Skinner MP, Fournier DJ, Andrews RK, Gorman JJ, Chesterman CN, Berndt MD. Characterization of human platelet GMP-140 as a heparin-binding protein. *Biochem Biophys Res Commun* 1989; **164**: 1373–1379.
54. Buckley TL, Brain SD, Collins PD, Williams TJ. Inflammatory edema induced by interactions between IL-1 and the neuropeptide calcitonin gene-related peptide. *J Immunol* 1991; **146**: 3424–3430.
55. Smith CH, Barker JNWN, Morris RW, MacDonald DM, Lee TH. Neuropeptides induce rapid expression of endothelial cell adhesion molecules and elicit granulocytic infiltration in human skin. *J Immunol* 1993; **151**: 3274–3282.
56. Ley K, Bulard DC, Arbonés ML, *et al.* Sequential contribution of L- and P-selectin to leukocyte rolling *in vivo*. *J Exp Med* 1995; **181**: 669–675.
57. Labow MA, Norton CR, Rumberger JM, *et al.* Characterization of E-selectin-deficient mice: demonstration of overlapping function of the endothelial selectins. *Immunity* 1994; **1**: 709–720.
58. Mulligan MS, Watson SR, Fennie C, Ward PA. Protective effects of selectin chimeras in neutrophil-mediated lung injury. *J Immunol* 1993; **151**: 6410–6417.
59. Bullard DC, Qin L, Lorenzo I, *et al.* P-selectin/ICAM-1 double mutant mice: acute emigration of neutrophils into the peritoneum is completely absent but is normal into pulmonary alveoli. *J Clin Invest* 1995; **95**: 1782–1788.
60. Mayadas TM, Johnson RC, Rayburn H, Hynes RO, Wagner DD. Leukocyte rolling and extravasation are severely compromised in P-selectin-deficient mice. *Cell* 1993; **74**: 541–554.
61. Tedder TF, Steeber DA, Pizcueta P. L-selectin-deficient mice have impaired leukocyte recruitment into inflammatory sites. *J Exp Med* 1995; **181**: 2259–2264.
62. Subramaniam M, Saffaripour S, Watson SR, Mayadas TN, Hynes RO, Wagner DD. Reduced recruitment of inflammatory cells in a contact hypersensitivity response in P-selectin-deficient mice. *J Exp Med* 1995; **181**: 2277–2282.
63. Wakelin MW, Sanz MJ, Albelda SA, Nourshargh S. An antibody recognizing rat PECAM-1 inhibits leukocyte extravasation across mesenteric microvessels *in vivo*. *Inflamm Res* 1995; **44**: S243.
64. Nygaard SD, Ganz T, Peterson MW. Defensins reduce the barrier integrity of a cultured epithelial monolayer without cytotoxicity. *Am J Respir Cell Mol Biol* 1993; **8**: 193–200.
65. Rosengren S, Arfors KE. Neutrophil-mediated vascular leakage is not suppressed by leukocyte elastase inhibitors. *Am J Physiol* 1990; **259**: H1288–H1294.

ACKNOWLEDGEMENTS. N.V.O. is a Senior Research Assistant of the Belgian Fund for Scientific Research (NFWO). This study was supported by the FGWO (grant 3.0068.94) and by the Belgian Programme on Interuniversity Poles of Attraction by the Belgian State, Prime Minister's Office, Science Policy Programming. The authors wish to thank L. Van den Eynde for typing the manuscript and A-E. Van Hoydonck for technical assistance.

**Received 1 August 1996; accepted 28 August 1996**
